# Monitored and Predicted Data for a Diesel Fuel Hydrotreating Reactor

**DOI:** 10.3390/ma18112481

**Published:** 2025-05-25

**Authors:** Laura Elisabeta Petraş, Tănase Dobre, Nela Şerbănescu, Florian Daniel Pop, Oana Cristina Pârvulescu

**Affiliations:** 1 Chemical and Biochemical Department, National University of Science and Technology POLITEHNICA Bucharest, 1-7 Gheorghe Polizu, 011061 Bucharest, Romania; laurapetras@yahoo.ro; 2 Petromidia Refinery, 214 Năvodari Bld., 905700 Năvodari, Romania; nela.serbanescu@rompetrol.com (N.Ş.); floriandaniel.pop@rompetrol.com (F.D.P.); 3 Technical Sciences Academy of Romania, 26 Dacia Bld., 030167 Bucharest, Romania

**Keywords:** diesel, hydrodenitrogenation, hydrodesulfurization, hydrotreating, power law kinetic model

## Abstract

A mathematical model based on power law kinetics was selected to simulate the hydrotreating process of diesel fuel (also called diesel oil or diesel), assuming hydrogenation reactions of sulfur compounds, nitrogen compounds, aromatic compounds, and olefins. The process efficiency depended on the diesel flow rate, catalyst volume, hydrogenation reaction rate constants, and reaction orders. The reaction rate constants were expressed as functions of the mean temperature in the catalyst bed using the Arrhenius equation. The parameters of the Arrhenius equation for the hydrogenation of sulfur and nitrogen compounds, i.e., the pre-exponential factors (6.515–15.62·10^4^ h^−1^) and activation energies (47.24–66.13 kJ/mol), were estimated based on monitoring data obtained in an industrial plant. The results obtained suggested that the catalyst used in the industrial reactor had almost equal specificity for the hydrogenation of sulfur and nitrogen compounds.

## 1. Introduction

Hydrotreating (HT) of oil products is a catalytic process in which the products are stabilized by hydrogenation of unsaturated hydrocarbons and/or removal of contaminants such as sulfur, nitrogen, oxygen, and metals (Ni, V) from feedstock by reacting them with hydrogen (H_2_), under relatively high temperature and pressure [1,2]. Several hydrogenation reactions occur simultaneously in the HT process, i.e., hydrodesulfurization (HDS), hydrodenitrogenation (HDN), hydrodearomatization (HDA), hydrodemetallization (HDM), hydrodeoxygenation (HDO), and olefin saturation (OS) [1,2,3]. Most crude oils contain low levels of oxygen, and consequently HDO is less of a concern. The HT process occurs as follows: a mixture of hydrocarbon feedstock and H_2_ is heated to a designed inlet temperature and then introduced into the catalytic reactor, loaded with a suitable catalyst, where the H_2_ reacts with the oil to produce mainly saturated hydrocarbons, hydrogen sulphide (H_2_S), and ammonia (NH_3_).

For the first time, HT was established as a process in the preprocessing of petroleum products for catalytic cracking in order to avoid the degradation of catalysts. Starting with the 1990s, HT turned into advanced HT, the goal being to have the content of sulfur compounds in fuels at the pump extremely low (below 10 ppm, according to the USA and EU regulations [4,5]). Advanced HT is essential for meeting strict air pollutant emission regulations.

**Figure 1:**
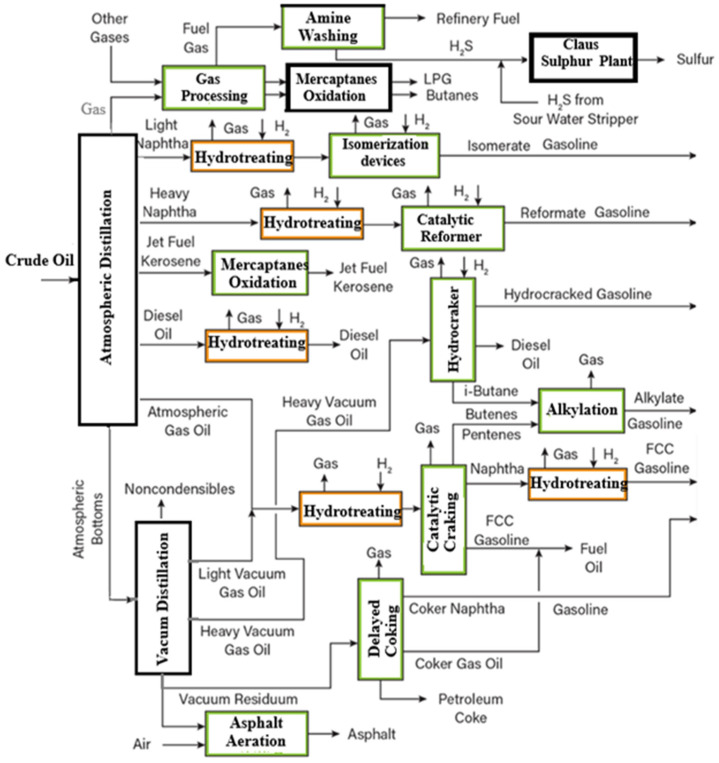
Process flow diagram of an oil refinery (adapted from [6]).

Five cases of HT, of which four are advanced HT, are reported in the crude oil processing technology presented in Figure 1, where the chemical processing of products from crude oil atmosphere distillation (AD) and vacuum distillation (VD) is marked [6]. The reactor effluent enters high-pressure and low-pressure separators where the liquid and gaseous products are separated. Depending on the case, the liquid product is sent to the fractionation units for further separation. Unreacted H_2_ is usually recovered from the gaseous product and recycled to the reactor with a small purge, which eliminates the possibility of accumulation of undesired compounds in the recycled H_2_ [7]. At most refineries, the mixture entering the HT reactor consists of diesel from the AD, light diesel from the VD, and kerosene from the AD (Figure 1). The concentrations of sulfur and nitrogen compounds in this mixture are generally 0.6–1 wt% and 0.1–0.3 wt%, respectively, those of aromatic compounds and olefins 26–35 wt%, and the mixture density typically ranges from 830 kg/m^3^ to 850 kg/m^3^.

**Figure 2:**
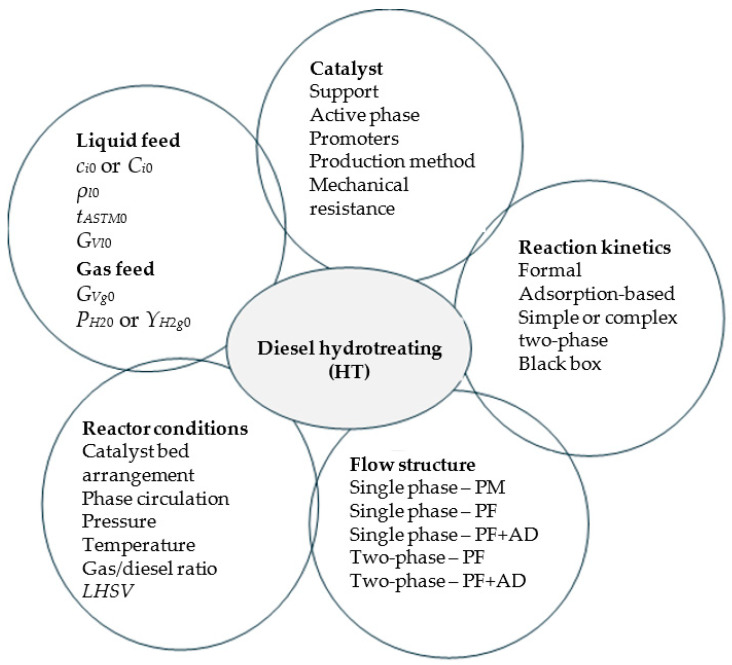
Main factors affecting the catalytic HT process of diesel fuel; *c_i_*_0_ or *C_i_*_0_, mass concentration or mass percentage of *S_i_* species (sulfur compounds, nitrogen compounds, aromatic compounds, and olefins) in the liquid feed (kg/m^3^ or wt%); *G_Vg_*_0_, volumetric flow rate of gas feed (m^3^/h); *G_Vl_*_0_, volumetric flow rate of liquid feed (m^3^/h); *LHSV*, liquid hourly space velocity (h^−1^); *P_H_*_20_, partial pressure of H_2_ in the gas feed (Pa); *t_ASTM_*_0_, ASTM 50% distillation temperature of liquid feed (°C); *Y_H_*_2*g*0_, molar percentage of H_2_ (H_2_ purity) in the gas feed (mol%); *ρ_l_*_0_, density of liquid feed (kg/m^3^); AD, axial dispersion; PF, plug flow; PM, perfect mixing.

The main factors affecting the HT process of diesel fuel are listed in Figure 2 [1,2,3,8,9]. Pressure (*P*), temperature (*t*), partial pressures of H_2_ in the gas feed (*P_H_*_20_) or molar percentage of H_2_ (H_2_ purity) in the gas feed (*Y_H_*_2*g*0_ = 100*P_H_*_20_/*P*), gas/diesel volumetric ratio (*r_gl_*), and liquid hourly space velocity (*LHSV*), i.e., the ratio of diesel volumetric flow rate to catalyst volume, are usually selected as quantitative independent variables (factors) of the HT process. The values of process factors depend on the type of the feedstock and the desired product specifications. The data summarized in Table 1 highlight that for low-boiling petroleum feedstock, e.g., naphtha, the levels of *P* and *t* in the ranges of 1.5–3.5 MPa and 260–340 °C, respectively, are sufficient for HT. However, for high-boiling petroleum feedstock, more severe conditions are required, i.e., 3.1–6.2 MPa and 330–410 °C, respectively.

A high pressure increases the solubility of H_2_ in the liquid phase, thus determining its better accessibility to the active centers of the catalyst. The levels of *t* are determined by the mechanism of catalytic processes specific to active catalytic centers, whose activation is also linked to *t*. The levels of *P_H_*_20_ and *r_gl_* are related, i.e., a low level of *P_H_*_20_ requires a high level of *r_gl_*. If the value of *r_gl_* is too low, a rapid deactivation of the catalyst occurs. As a rule, the minimum level of *r_gl_* should be at least five times the amount of H_2_ consumed in the characteristic chemical reactions of the HT process [15]. For HDS and HDN, there is an optimal value for *r_gl_*, which depends on the nature of the feedstock and operating conditions [16]. Unreacted H_2_ is recovered from the reactor effluent and recycled to the hydrotreater. Purging a small part of the gas effluent, enriching it in H_2_ through a suitable technological solution (e.g., membrane separation), and supplementing H_2_ as pure as possible, lead to maintaining the purity of H_2_ entering the reactor at over 90 mol%. At H_2_ purities lower than those specified in Table 1, achieving advanced HT requires an increase in *t*, resulting in a faster deactivation of the catalyst.

Regarding the effect of H_2_ purity on HDS, HDN, and HDA, a study on the HT of a vacuum gas oil (VGO) Arabian Light (with the following levels of sulfur, nitrogen, and aromatic compound contents at the reactor inlet: *C_S_*_0_ = 2.430 wt% = 24,300 ppm, *C_N_*_0_ = 0.065 wt% = 650 ppm, and *C_A_*_0_ = 47 wt%) reported that the sulfur, nitrogen, and aromatic compound contents of the hydrotreated VGO (*C_Se_*, *C_Ne_*, and *C_Ae_*) decreased as *P_H_*_20_ increased from 70 to 140 bar, i.e., *C_Se_* decreased from 200 to 40 ppm, *C_Ne_* from 10 to 1 ppm, and *C_Ae_* from 31 to 9.6 wt% [17]. This indicates that HDN was more sensitive than HDS and HDA to the partial pressure of H_2_.

The type of catalyst is a qualitative factor that can significantly improve the process performance [18,19,20,21]. The catalysts widely used in the HT process are synthesized in the form of oxide on Al_2_O_3_ support and then activated by converting them into sulfide form through a sulfidation procedure [18]. The active phase for the HT catalysts used in industrial reactors is usually CoMoS or NiMoS [18]. The key element for the catalyst activity is the concept of vacancy [19,21]. When H_2_ reacts with a surface sulfide group, H_2_S is released and an S vacancy is created. The size and shape of the catalyst as well as its wettability by the processed liquid are of great interest because they determine the phase flow structure and the value of the solid–liquid or gas–liquid specific surface area. Moreover, the catalyst particles should have a good mechanical resistance.

Modelling is a valuable tool in the design, control, and optimization of chemical processes [22,23]. A dynamic or stationary mathematical model for a HT reactor should contain the equations for [23,24,25,26,27,28,29,30]: (i) species mass balance in the liquid and gas phases (depending on flow structure); (ii) catalytic process kinetics for all species; (iii) interphase equilibrium for the species that perform interphase transfer; (iv) species transfer kinetics at the solid–liquid and gas–liquid interfaces.

Global kinetic (GK) models, including the power law (PL), Langmuir–Hinshelwood (L–H), and multi-parameter (MP) kinetic models, are widely used to predict the hydrogenation reaction rates [31,32,33,34,35,36,37,38,39,40,41,42,43,44,45,46]. GK models for a HT reactor are based on the following simplifying assumptions: (i) stationary state; (ii) plug flow of the gas phase; (iii) a large excess of H_2_ in the gas phase compared to the stoichiometry of the chemical reactions; (iv) perfectly mixed liquid phase; (v) the pressure and purity of H_2_ in the gas phase are high enough so that its concentration in the liquid phase is close to the saturation concentration; and (vi) the interphase transfer of any species is much faster compared to the chemical reactions.

In a PL model, the rates of catalytic chemical reactions are expressed by power law relationships. L–H models are characterized by the fact that the expression for the reaction rate contains, in order to take into account the catalyst inhibition, the species adsorption on the catalyst surface [32,33,34,36,39]. MP models are similar to PL models but take into account the effects of more variables on the hydrotreating process, e.g., *P_H_*_2_, *r_gl_* [32].

In our previous paper [41], a simple model based on PL kinetics and reaction order (*n_i_*) of 1 for the hydrogenation of *S_i_* species (sulfur compounds, nitrogen compounds, aromatic compounds, and olefins) was used to predict the performance of diesel HT. The model parameters, namely the activation energies for hydrogenation reactions and pre-exponential factors in the Arrhenius equation (*E_hi_* and *k_hi_*_0_), were selected from data reported in the literature. In this paper, the previous model was developed, and its adjustable parameters, i.e., *E_hi_*, *k_hi_*_0_, and *n_i_*, were estimated based on monitoring data obtained in an industrial plant. The model could be useful for industrial process control and optimization of relevant factors.

## 2. Materials and Methods

### 2.1. Modelling

PL kinetic models assume that the hydrogenation mechanism of *S_i_* species is described by an equilibrium reaction expressed by Equation (1), where *k_hi_* (h^−1^) is the hydrogenation reaction rate constant and *k_dhi_* (h^−1^) the dehydrogenation reaction rate constant.

(1) $$S_{i}+a_{i}H_{2}\overset{k_{hi}}{\underset{k_{dhi}}{\Leftrightarrow }}S_{i}{\left(H_{2}\right)}_{a_{i}}\Rightarrow \text{Products}$$

Assuming *k_hi_* >> *k_dhi_* and an excess of H_2_, then the chemical reaction rate of *S_i_* species (*v_ri_*) can be described by Equation (2), where *c_i_* (kg/m^3^) represents the mass concentration of *S_i_* species in the liquid phase, *τ* (h) the time, and *n_i_* the reaction order. For HDS and HDN, this assumption is valid at *t* > 360 °C.

(2) $$v_{ri}=-\frac{dc_{i}}{d\tau }=k_{hi}{c_{i}}^{n_{i}}$$

**Figure 3:**
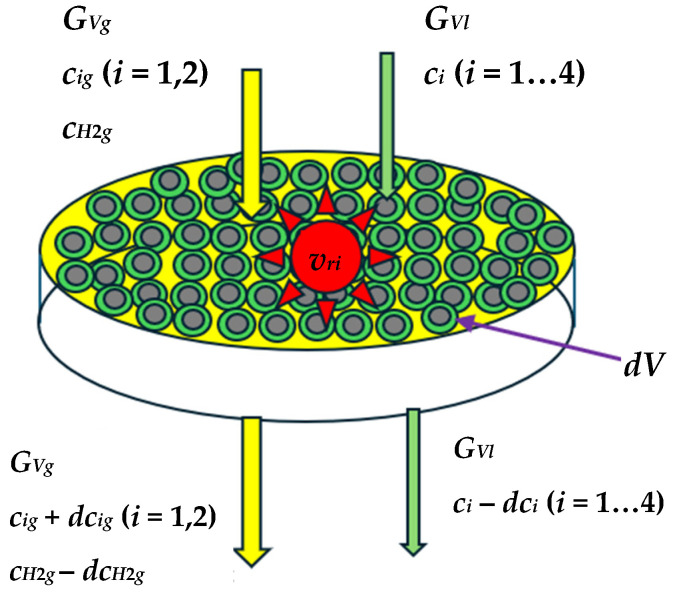
Schema for species mass balance for a control volume in the HT reactor.

The mass balances of *S_i_* species in the liquid and gas phases are given by Equations (3) and (4), where *c_ig_* (kg/m^3^) represents the mass concentration of *S_i_* species in the gas phase, *dV* (m^3^) is the elementary volume of the catalyst (Figure 3), *G_Vl_* and *G_Vg_* are the volumetric flow rates (m^3^/h) of liquid and gas phases, *α*_1_ is the stoichiometric ratio between hydrogen sulfide (H_2_S) and sulfur compounds, and *α*_2_ the stoichiometric ratio between ammonia (NH_3_) and nitrogen compounds.

(3) $$G_{Vl}dc_{i}=-k_{hi}{c_{i}}^{n_{i}}dV,i=1\ldots 4$$

(4) $$G_{Vg}dc_{ig}=\alpha_{i}k_{hi}{c_{i}}^{n_{i}}dV,i=1,2$$

Differential Equations (5) and (6) were obtained by rearranging the terms in Equations (3) and (4). The mass balance of *H*_2_ in the gas phase is expressed by Equation (7), where *c_H_*_2*g*_ (kg/m^3^) is the mass concentration of *H*_2_ in the gas phase and *β_i_* (*i* = 1…4) represent the stoichiometric ratios between *H*_2_ and different species, i.e., sulfur compounds (*β*_1_), nitrogen compounds (*β*_2_), aromatic compounds (*β*_3_), and olefins (*β*_4_).

(5) $$\frac{dc_{i}}{dV}=-\frac{k_{hi}}{G_{Vl}}{c_{i}}^{n_{i}},i=1\ldots 4$$

(6) $$\frac{dc_{ig}}{dV}=\frac{\alpha_{i}k_{hi}}{G_{Vg}}{c_{i}}^{n_{i}},i=1,2$$

(7) $$\frac{dc_{H2g}}{dV}=-\sum_{i=1}^{4}\frac{\beta_{i}k_{hi}}{G_{Vg}}{c_{i}}^{n_{i}}$$

For a perfectly mixed liquid phase and low concentrations of the target compounds in the hydrogenation process, i.e., sulfur compounds, nitrogen compounds, aromatic compounds, and olefins, Equation (5) can be integrated analytically. Accordingly, taking into account the boundary condition expressed by Equation (8), the solutions of Equation (5) are given by Equations (9) and (10) for *n_i_* = 1 and *n_i_* ≠ 1, respectively, where *τ_LHSV_* = 1/*LHSV* (h) represents the residence time of the liquid phase in the catalyst bed, *V* (m^3^) is the catalyst volume, and *c_i_*_0_, *c_ig_*_0_, and *c_H_*_2*g*0_ are the mass concentrations (kg/m^3^) of *S_i_* species and *H*_2_ at *V* = 0.

(8) $$V=0,\;c_{i}=c_{i0}\;\left(i=1...4\right),\;c_{ig}=c_{ig0}=0\;\left(i=1,2\right),\;c_{H2g}=c_{H2g0}$$

(9) $$\frac{c_{i}}{c_{i0}}=\exp\left(-\frac{k_{hi}}{G_{Vl}}V\right)=\exp\left(-k_{hi}\tau_{LHSV}\right),i=1\ldots 4,n_{i}=1$$

(10) $$\frac{c_{i}}{c_{i0}}=\frac{1}{{\left[1+\frac{{c_{i}}^{n_{i}-1}\left(n_{i}-1\right)k_{hi}}{G_{Vl}}V\right]}^{\frac{1}{n_{i}-1}}}=\frac{1}{{\left[1+{c_{i}}^{n_{i}-1}\left(n_{i}-1\right)k_{hi}\tau_{LHSV}\right]}^{\frac{1}{n_{i}-1}}},i=1\ldots 4,n_{i}\neq 1$$

Hydrogenation degrees of *S_i_* species (*η_i_*) are expressed depending on the *c_i_*/*c_i_*_0_ ratio using Equations (11) and (12) for *n_i_* = 1 and *n_i_* ≠ 1, respectively.

(11) $$\eta_{i}=1-\frac{c_{i}}{c_{i0}}=1-\exp\left(-k_{hi}\tau_{LHSV}\right),i=1\ldots 4,n_{i}=1$$

(12) $$\eta_{i}=1-\frac{c_{i}}{c_{i0}}=1-\frac{1}{{\left[1+{c_{i}}^{n_{i}-1}\left(n_{i}-1\right)k_{hi}\tau_{LHSV}\right]}^{\frac{1}{n_{i}-1}}},i=1\ldots 4,n_{i}\neq 1$$

The hydrogenation reaction rate constant of *S_i_* species (*k_hi_*) can be expressed depending on the mean absolute temperature in the catalyst bed (*T_m.bed_*) using the Arrhenius Equation (13), where *E_hi_* (kJ/mol) represents the activation energy for the hydrogenation reaction, *k_hi_*_0_ (h^−1^) the pre-exponential factor, and *R* (kJ/molK) the universal gas constant. Equations (14) and (15) were obtained by substituting Equation (13) into Equations (9) and (10).

(13) $$k_{hi}=k_{hi0}\exp\left(-\frac{E_{hi}}{RT_{m,bed}}\right)$$

(14) $$\frac{c_{i}}{c_{i0}}=\exp\left[-\frac{Vk_{hi0}\exp\left(-\frac{E_{hi}}{RT_{m,bed}}\right)}{G_{Vl}}\right],i=1\ldots 4,n_{i}=1$$

(15) $$\frac{c_{i}}{c_{i0}}=\frac{1}{{\left[1+\frac{{c_{i}}^{n_{i}-1}\left(n_{i}-1\right)Vk_{hi0}\exp\left(-\frac{E_{hi}}{RT_{m,bed}}\right)}{G_{Vl}}\right]}^{\frac{1}{n_{i}-1}}},i=1\ldots 4,n_{i}\neq 1$$

### 2.2. Experimental

**Figure 4:**
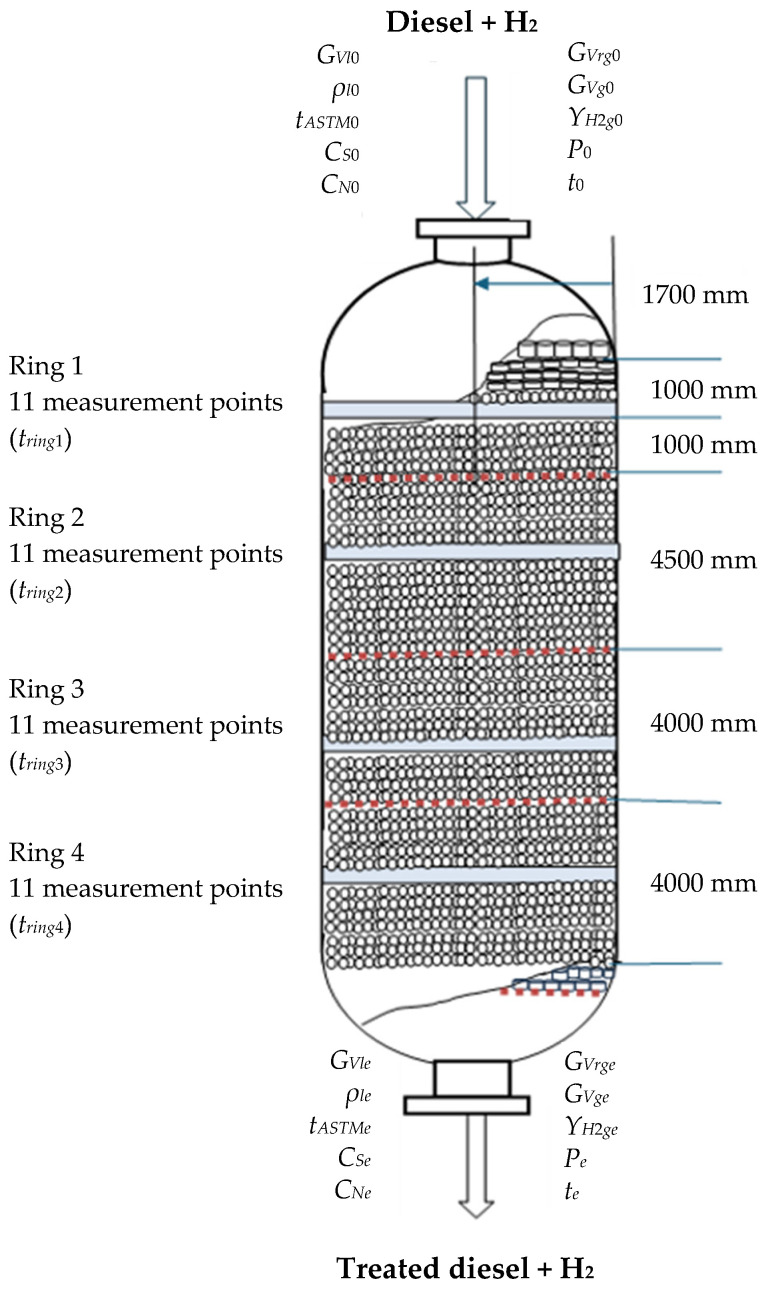
Dimensional schema of the industrial reactor and monitored variables.

Data from a 50-day monitoring set of a reactor in a diesel HT plant with a mean capacity of 100 m^3^/h diesel were provided by Petromidia Refinery (Năvodari, Romania). A schema of the HT reactor, including some geometric dimensions and monitored process variables, is shown in Figure 4. The following variables were measured at the inlet (0) and outlet (e) of the reactor: diesel volumetric flow rate (*G_Vl_*), diesel density (*ρ_l_*), diesel ASTM 50% distillation temperature (*t_ASTM_*), diesel sulfur and nitrogen mass percentages (*C_S_* = 100*c_S_*/*ρ_l_* and *C_N_* = 100*c_N_*/*ρ_l_*), volumetric flow rate of recycled gas (*G_Vrg_*), total volumetric flow rate of gas phase (*G_Vg_*), H_2_ purity in the gas phase (*Y_H_*_2*g*_ = 100*P_H_*_2_/*P*), pressure (*P*), and temperature (*t*). Moreover, temperatures in the areas of rings 1–4 (11 measurements points for each ring), i.e., *t_ring_*_1_, *t_ring_*_2_, *t_ring_*_3_, and *t_ring_*_4_, were monitored.

### 2.3. Data Processing

The data were processed using Mathcad 14 (PTC, Boston, MA, USA).

## 3. Results and Discussion

### 3.1. Monitored and Predicted Dynamics of Sulfur and Nitrogen Compound Concentrations in Diesel Fuel at the Reactor Outlet

**Figure 5:**
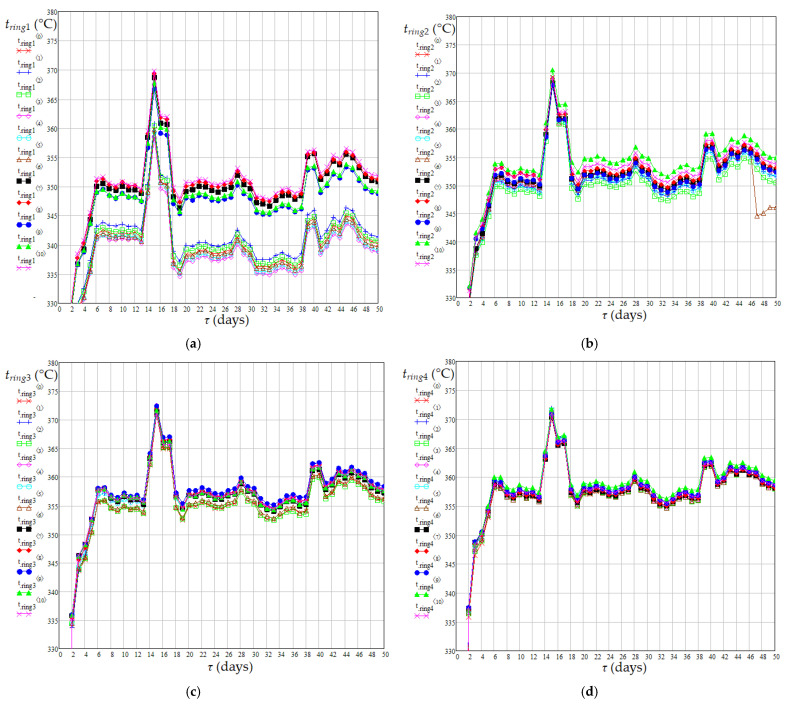
Dynamics of catalyst bed temperature (°C) at 11 measurement points in the area of: ring 1 (**a**); ring 2 (**b**); ring 3 (**c**); ring 4 (**d**).

**Figure 6:**
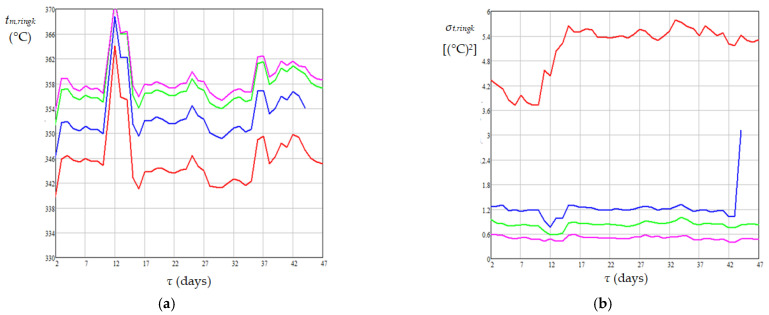
Dynamics of mean temperatures (**a**) and related dispersions (**b**) for ring *k* (*k* = 1 in red; *k* = 2 in blue; *k* = 3 in green; *k* = 4 in pink).

The results of temperature monitoring for 50 days at 11 measurement points in the areas of rings 1–4 are shown in Figure 5. Mean values of daily temperatures obtained from 11 measurements for each ring and related dispersions of temperature values around the mean values are shown in Figure 6.

For a model with *n_i_* = 1 (*i* = 1,2), the identification of the adjustable parameters (*k_hi_*_0_ and *E_hi_*) is based on the minimization of the objective functions *F*(*k_hi_*_0_, *E_hi_*) and *G*(*k_hi_*_0_, *E_hi_*) given by Equations (16) and (17), where *G_Vl_* = *G_Vl_*_0_ = *G_Vle_*. These objective functions represent the sum of squares of the residuals, i.e., differences between the monitored and predicted values of the concentrations of sulfur and nitrogen compounds at the reactor outlet. For a model with *n_i_* ≠ 1 (*i* = 1,2), the objective functions *F*(*n_i_*, *k_hi_*_0_, *E_hi_*) and *G*(*n_i_*, *k_hi_*_0_, *E_hi_*) are expressed by Equations (18) and (19).

(16) $$F\left(k_{hS0},E_{hS}\right)=\sum_{j=1}^{50}{\left\{c_{Sej}-c_{S0j}\exp\left[\frac{-k_{hS0}V\exp\left(-\frac{E_{hS}}{RT_{j,m,bed}}\right)}{G_{Vlj}}\right]\right\}}^{2},n_{S}\neq 1$$

(17) $$G\left(k_{hN0},E_{hN}\right)=\sum_{j=1}^{50}{\left\{c_{Nej}-c_{N0j}\exp\left[\frac{-k_{hN0}V\exp\left(-\frac{E_{hN}}{RT_{j,m,bed}}\right)}{G_{Vlj}}\right]\right\}}^{2},n_{N}=1$$

(18) $$F\left(n_{S},k_{hS0},E_{hS}\right)=\sum_{j=1}^{50}{\left\{c_{Sej}-\frac{c_{S0j}}{{\left[1+\frac{{c_{Sej}}^{n_{S}-1}\left(n_{S}-1\right)k_{hS0}V\exp\left(-\frac{E_{hS}}{RT_{j,m,bed}}\right)}{G_{Vlj}}\right]}^{\frac{1}{n_{S}-1}}}\right\}}^{2},n_{S}\neq 1$$

(19) $$G\left(n_{N},k_{hN0},E_{hN}\right)=\sum_{j=1}^{50}{\left\{c_{Nej}-\frac{c_{N0j}}{{\left[1+\frac{{c_{Nej}}^{n_{N}-1}\left(n_{N}-1\right)k_{hN0}V\exp\left(-\frac{E_{hN}}{RT_{j,m,bed}}\right)}{G_{Vlj}}\right]}^{\frac{1}{n_{N}-1}}}\right\}}^{2},n_{N}\neq 1$$

**Figure 7:**
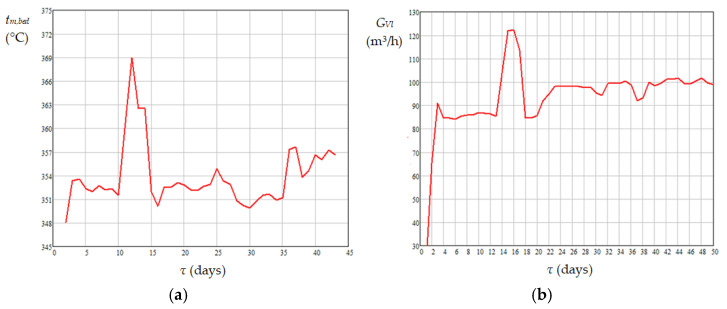
Dynamics of mean temperature in the catalyst bed (**a**) and diesel flow rate through the industrial HT reactor (**b**).

**Figure 8:**
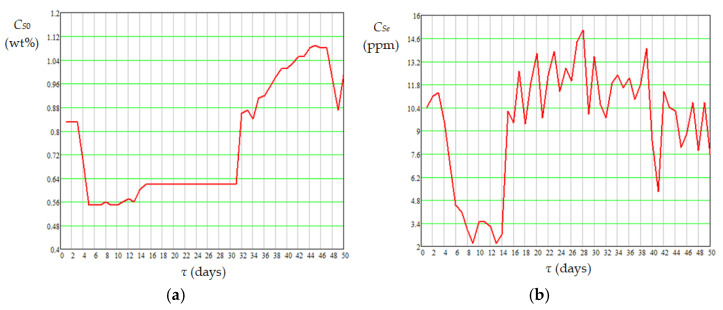
Dynamics of sulfur compound concentration in diesel fuel at the reactor inlet (**a**) and outlet (**b**).

**Figure 9:**
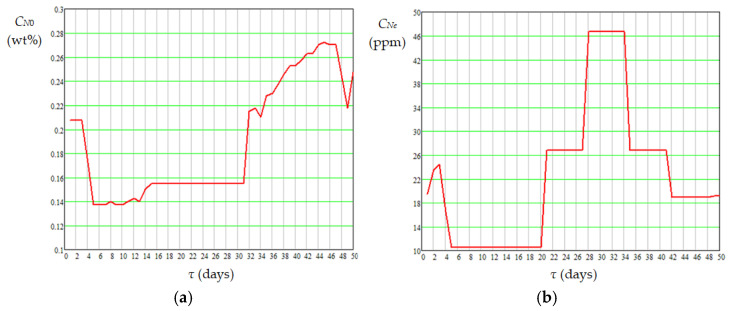
Dynamics of nitrogen compound concentration in diesel fuel at the reactor inlet (**a**) and outlet (**b**).

The mean values of absolute daily temperature in the catalyst bed appearing in Equations (16)–(19), i.e., *T_j_*_,*m*,*bed*_ (K), were calculated using Equation (20). Dynamics of mean temperature in the catalyst bed (*t_m_*_,*bed*_), diesel flow rate at the reactor inlet (*G_Vl_*), mass percentages of sulfur and nitrogen compounds in diesel fuel at the reactor inlet (*C_S_*_0_ and *C_N_*_0_) and outlet (*C_Se_* and *C_Ne_*) are shown in Figure 7, Figure 8 and Figure 9. Data presented in Figure 5, Figure 6, Figure 7, Figure 8 and Figure 9 highlight the following: (i) the approach to the steady state of the HT installation took several days and consisted in bringing the catalyst into the thermal operating regime (Figure 5, Figure 6, and Figure 7a) simultaneously with the increase in *G_Vl_* to around 100 m^3^/h (Figure 7b); (ii) variations in the independent variables, including *t_m_*_,*bed*_ (Figure 7), *C_S_*_0_ (Figure 8a), and *C_N_*_0_ (Figure 9a), led to variations in the mass percentages of sulfur and nitrogen compounds in the diesel fuel at the reactor outlet, i.e., *C_Se_* (Figure 8b) and *C_Ne_* (Figure 9b); (iii) *G_Vl_* increased significantly on days 13–15, this increase being imposed by a significant increase in *t_m_*_,*bed*_ on days 10–12 (Figure 7); (iv) the mean temperature measured in the area of ring 4 (near the reactor exit) was higher than that measured in the area of ring 1 (near the reactor entrance), the mean difference between them being of about 14 °C (Figure 6a); (v) the mean temperature measured in the area of ring 1 had a much higher dispersion than those measured in the areas of rings 2–4 (Figure 6b); (vi) the dynamics of nitrogen compound concentration in the diesel fuel at the reactor inlet (Figure 9a) and outlet (Figure 9b) suggest that between days 20 and 42 the catalyst activity for nitrogen compound hydrogenation decreased.

(20) $$T_{j,m,bed}=273.15+t_{j,m,bed}=273.15+\frac{\sum_{k=1}^{4}t_{j,m,ringk}}{4},j=1\ldots 50$$

For sulfur compounds, the data were processed using Mathcad 14 in the following order: (i) import of the file with the monitored data (Table 2); (ii) the choice based on literature data [34,35,41,42,47,48,49] of the ranges of variation of the parameters of the Arrhenius Equation (13), i.e., 3.6–5.0·10^5^ h^−1^ for *k_hS_*_0_ and 52–64 kJ/mol for *E_hS_*; (iii) determining the values of the objective function *F*(*k_hS_*_0_,*E_hS_*) given by Equation (16) at different levels of *k_hS_*_0_ and *E_hS_* (specified in Table 3), and identifying the parameter levels that lead to a minimum value of the objective function, i.e., *k_hS_*_0,*min*,*I*_ = 4.0·10^5^ h^−1^ and *E_hS_*_,*min*,*I*_ = 56 kJ/mol, for which *F_min_*_,*I*_ = 1.702·10^3^ kg^2^/m^6^ (Table 3); (iv) identifying the parameter levels to obtain the minimum value of the objective function by solving the system of Equations (21), where *k_hS_*_0,*min*,*I*_ and *E_hS_*_,*min*,*I*_ were used as initial values, i.e., *k_hS_*_0,*min*,*II*_ = 8.189·10^4^ h^−1^ and *E_hS_*_,*min*,*II*_ = 47.75 kJ/mol, for which *F_min_*_,*II*_ = 1.641·10^3^ kg^2^/m^6^. The same procedure was applied for data processing for nitrogen compounds. The values of adjustable parameters for HDS and HDN identified from monitoring data using Equations (16)–(19), including *k_hS_*_0,*min*,*II*_ = *k_hS_*_0_, *E_hS_*_,*min*,*II*_ = *E_hS_*, *k_hN_*_0,*min*,*II*_ = *k_hN_*_0_, *E_hN_*_,*min*,*II*_ = *E_hN_*, *n_S_*, and *n_N_*, are summarized in Table 4. Tabulated data indicate the following aspects: (i) the values of *k_hS_*_0_, *E_hS_*, *k_hN_*_0_, and *E_hN_* are quite close to other data reported in the literature [34,35,41,42,47,48,49]; (ii) the values of *k_hS_*_0_ and *k_hN_*_0_ and those of *E_hS_* and *E_hN_* for *n_i_* = 1 were extremely close, suggesting that the type of catalyst used in the reactor had almost equal specificity for the hydrogenation of sulfur and nitrogen compounds; (iii) the results obtained for *n_i_* ≠ 1, i.e., very close values of *n_S_* and *n_N_*, *k_hS_*_0_ and *k_hS_*_0_, and *E_hS_* and *E_hN_*, respectively, also support the hypothesis of equal specificity of the catalyst towards sulfur and nitrogen compounds.

(21) $$\left\{\begin{array}{c} \frac{\partial F\left(k_{hS0},E_{hS}\right)}{\partial k_{hS0}}=0\\ \frac{\partial F\left(k_{hS0},E_{hS}\right)}{\partial E_{hS}}=0 \end{array}\right.$$

In addition, the values of the Arrhenius equation parameters can provide valuable information about the reduction in catalyst activity over time. For example, if the values of *E_hS_* and *E_hN_* are determined from monitoring data with a certain frequency (e.g., monthly, every two months), the degree of catalyst deactivation can be assessed based on the variation over time of these parameter values (the lower the values, the greater the catalyst deactivation).

**Figure 10:**
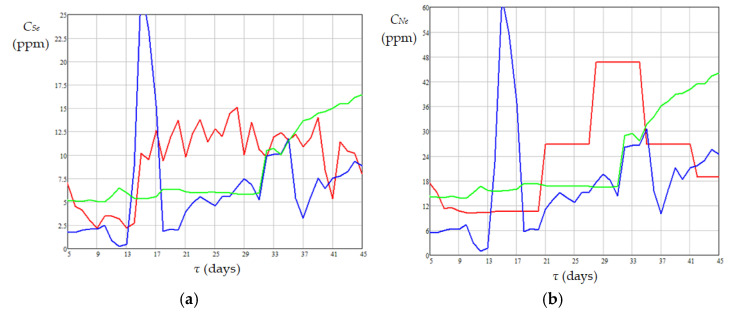
Dynamics of sulfur compound concentrations (**a**) and nitrogen compound concentrations (**b**) in diesel fuel at the reactor outlet: monitored values in red; predicted values in blue (*n_i_* = 1) and green (*n_i_* ≠ 1), respectively (based on data summarized in Table 4).

Dynamics of experimental (monitored) and predicted concentrations of sulfur and nitrogen compounds in diesel fuel at the reactor outlet, which are shown in Figure 10, highlight that the data predicted by Equations (14) and (15) cover the experimental data quite well. Moreover, a significant increase in the predicted concentrations of sulfur and nitrogen compounds for *n_i_* = 1 is observed on days 13–15, this increase also occurring during this period for the diesel flow rate (Figure 7).

Many other monitoring data, showing the dynamics of other independent and dependent variables, may be of interest for models that take into account a more complex phenomenology of HT. Accordingly, the monitoring data can be supplemented with the dynamics of: (i) volumetric flow rate of recirculated gas at the reactor inlet (*G_Vrg_*_0_) (Figure S1a) and H_2_ purity of recirculated gas (*Y_H_*_2*rg*0_) (Figure S1b), (ii) volumetric flow rate of fresh gas at the reactor inlet (*G_Vfg_*_0_) (Figure S2a) and H_2_ purity of fresh gas (*Y_H_*_2*fg*0_) (Figure S2b), (iii) diesel density at the reactor inlet and outlet (*ρ_l_*_0_ and *ρ_le_*) (Figure S3a), and (iv) diesel ASTM 50% distillation temperature at the reactor inlet and outlet (*t_ASTM_*_0_ and *t_ASTMe_*) (Figure S3b).

### 3.2. Predicted Dynamics of Compound Concentrations in Diesel Fuel at Different Levels of Process Factors

**Figure 11:**
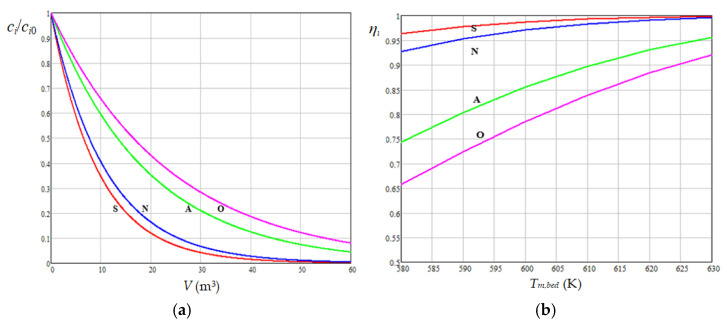
Dimensionless species concentrations vs. catalyst volume at 600 K (**a**) and species hydrogenation degrees vs. mean temperature in the catalyst bed at *V* = 60 m^3^ (*LHSV* = 1.67 h^−1^) (**b**); S, sulfur compounds; N, nitrogen compounds; A, aromatic compounds; O, olefins; *G_Vl_* = 100 m^3^/h; *n_S_* = *n_N_* = *n_A_* = *n_O_* = 1; *k_hS_*_0_ = 2.085·10^4^ h^−1^; *k_hN_*_0_ = 4.285·10^4^ h^−1^; *k_hA_*_0_ = 8.061·10^4^ h^−1^; *k_hO_*_0_ = 8.842·10^4^ h^−1^; *E_hS_* = 39.70 kJ/mol; *E_hN_* = 44.30 kJ/mol; *E_hA_* = 50.50 kJ/mol; *E_hO_* = 52.10 kJ/mol.

Predicted curves of dimensionless species concentrations (*c_i_*/*c_i_*_0_ ≈ *C_i_*/*C_i_*_0_, *i* = 1…4) depending on catalyst volume (*V*) at *T_m_*_,*bed*_ = 600 K are shown in Figure 11a. The curves were predicted using the model with first-order kinetics (*n_i_* = 1) described by Equation (14), where *G_Vl_* = 100 m^3^/h and the kinetic parameters of the Arrhenius equation (*k_hi_*_0_ and *E_hi_*) were selected based on data reported in the related literature [34,35,41,42,47,48,49]. The results presented in Figure 11a indicate that to reduce the sulfur concentration of diesel fuel from 6000 ppm to 6 ppm, a catalyst volume of 60 m^3^ is required, which is in agreement with the values of *V* corresponding to an industrial reactor. Predicted curves of species hydrogenation degrees (*η_i_* = 1 − *c_i_*/*c_i_*_0_, *i* = 1…4) depending on *T_m_*_,*bed*_ at *V* = 60 m^3^ (*LHSV* = 1.67 h^−1^) (Figure 11b) highlight a higher sensitivity to *T_m_*_,*bed*_ of the hydrogenation of olefins, aromatic and nitrogen compounds compared to that of sulfur compounds. Equation (14) indicates an increase in *c_i_*/*c_i_*_0_ with an increase in *G_Vl_* and a decrease in *V* and *T_m_*_,*bed*_, consistent with the variations of *c_i_*/*c_i_*_0_ and *η_i_* with the process factors that are presented in Figure 11.

## 4. Conclusions

Assuming that the HT process of diesel fuel is controlled by the kinetics of hydrogenation reactions of sulfur compounds, nitrogen compounds, aromatic compounds, and olefins, a PL kinetic model was selected to predict the hydrogenation reaction rates. According to this model, the species dimensionless mass concentrations (*c_i_*/*c_i_*_0_, *i* = 1…4) were functions of diesel flow rate (*G_Vl_*), catalyst volume (*V*), hydrogenation reaction rate constants (*k_hi_*), and reaction orders (*n_i_*). The Arrhenius equation was used to express the dependence between *k_hi_* and mean absolute temperature in the catalyst bed (*T_m_*_,*bed*_).

Predicted curves of *c_i_*/*c_i_*_0_ depending on *V* at *T_m_*_,*bed*_ = 600 K, where *G_Vl_* = 100 m^3^/h, *n_i_* = 1, and the kinetic parameters of the Arrhenius equation (*k_hi_*_0_ and *E_hi_*) were selected based on data from the literature, indicated a catalyst volume of 60 m^3^ (*LHSV* = 1.67 h^−1^) required to reduce the sulfur concentration of diesel fuel from 6000 ppm to 6 ppm. This value of *V* is in agreement with those corresponding to an industrial reactor. Predicted curves of species hydrogenation degrees (*η_i_* = 1 − *c_i_*/*c_i_*_0_) depending on *T_m_*_,*bed*_ at *V* = 60 m^3^ revealed a higher sensitivity to *T_m_*_,*bed*_ of the hydrogenation of olefins, aromatic and nitrogen compounds compared to that of sulfur compounds.

Data measured over 50 days in an industrial plant were provided. The model adjustable parameters in terms of kinetic parameters of the Arrhenius equation were estimated by minimizing the sum of squares of the differences between the monitored and predicted values of the concentrations of sulfur and nitrogen compounds at the reactor outlet. The results obtained, i.e., very close values of the kinetic parameters, suggested that the catalyst had almost equal specificity for the hydrogenation of sulfur and nitrogen compounds.

Consequently, a simple model based on PL kinetics was used to predict the performance of the diesel HT process under various operating conditions. An optimization study could be performed for the levels of process factors used in an industrial reactor, typically *G_Vl_* = 80–120 m^3^/h, *V* = 50–150 m^3^, and *T_m_*_,*bed*_ = 603–653 K (*t_m_*_,*bed*_ = 330–380 °C). In addition, it is necessary to consider how the activation energies for hydrogenation reactions of *S_i_* species evolve so that the reduction in catalyst activity can be correctly assessed. These aspects will be addressed in future work.

## Figures and Tables

**Table 1:** Ranges of values of HT factors.

Case	Pressure (MPa)	Temperature (°C)	H_2_ Purity (mol%)	Gas/Diesel Ratio (m^3^/m^3^)	Catalyst Type	Ref.
Light diesel	1.5–3.5	260–340	86–96	350–1000	CoMoS or NiMoS	[10,11,12]
Heavy diesel	3.1–6.2	330–410	88–96	400–1100	CoMoS or NiMoS	[13,14]

**Table 2:** Sequence from the file containing the monitoring data.

Day	*G_Vl_* (m^3^/h)	*t_m_*_,*bed*_ (°C)	*C_S_*_0_ (wt%)	*C_Se_* (wt%)
1	20.1	335	0.83	1.04·10^−3^
2	65.3	348	0.83	1.11·10^−3^
3	90.9	353	0.69	1.13·10^−3^
4	84.6	354	0.55	0.93·10^−3^
^..........^	^..........^	^..........^	^..........^	^..........^
47	101.8	355	0.98	0.78·10^−3^
48	99.6	354	0.87	1.07·10^−3^
49	98.6	352	0.99	0.76·10^−3^
50	84.7	355	0.96	1.44·10^−3^

**Table 3:** Values of *F*(*k_hS_*_0_,*E_hS_*) calculated using Equation (16) at different levels of *k_hS_*_0_ and *E_hS_*.

	*E_hS_* (kJ/mol)	52	54	56	58	60	62	64
*k_hS_*_0_ (h^−1^)								
**3.6·10^5^**		4.272·10^3^	3.669·10^3^	4.076·10^3^	3.871·10^5^	6.235·10^6^	4.066·10^7^	1.470·10^8^
**3.8·10^5^**		4.278·10^3^	3.891·10^3^	2.140·10^3^	2.354·10^5^	4.499·10^6^	3.254·10^7^	1.261·10^8^
**4.0·10^5^**		4.282·10^3^	4.035·10^3^	1.702·10^3^	1.419·10^5^	3.245·10^6^	2.604·10^7^	1.082·10^8^
**4.2·10^5^**		4.283·10^3^	4.127·10^3^	1.881·10^3^	8.464·10^4^	2.340·10^6^	2.084·10^7^	9.291·10^7^
**4.4·10^5^**		4.284·10^3^	4.185·10^3^	2.267·10^3^	4.983·10^4^	1.686·10^6^	1.669·10^7^	7.977·10^7^
**4.6·10^5^**		4.285·10^3^	4.222·10^3^	2.678·10^3^	2.887·10^4^	1.214·10^6^	1.336·10^7^	6.849·10^7^
**4.8·10^5^**		4.285·10^3^	4.245·10^3^	3.045·10^3^	1.645·10^4^	8.725·10^5^	1.070·10^7^	5.881·10^7^
**5.0·10^5^**		4.285·10^3^	4.260·10^3^	3.346·10^3^	9.253·10^3^	6.262·10^5^	8.568·10^6^	5.051·10^7^

**Table 4:** Model parameters for HDS and HDN identified from monitoring data using Equations (16)–(19).

*n_S_*	*k_hS_*_0_ (h^−1^)	*E_hS_* (kJ/mol)	*n_N_*	*k_hN_*_0_ (h^−1^)	*E_hN_* (kJ/mol)
1	8.189·10^4^	47.75	1	6.515·10^4^	47.24
0.914	1.192·10^5^	62.91	0.908	1.562·10^5^	66.13

## Data Availability

The original contributions presented in this study are included in the article and Supplementary Materials. Further inquiries can be directed to the corresponding authors.

## Supplementary Materials

The following supporting information can be downloaded at: https://www.mdpi.com/article/10.3390/ma18112481/s1, Figure S1: Dynamics of volumetric flow rate of recirculated gas at the reactor inlet (a) and hydrogen purity of recirculated gas (b); Figure S2: Dynamics of volumetric flow rate of fresh gas at the reactor inlet (a) and hydrogen purity of fresh gas (b); Figure S3: Dynamics of diesel density (a) at the reactor inlet (in red) and outlet (in blue) and ASTM 50% distillation temperature (b) at the reactor inlet (in red) and outlet (in blue).

## Abbreviations

*c_i_*	mass concentration of *S_i_* species in the liquid phase, kg/m^3^
*c_H_*_2_ *_g_*	mass concentration of hydrogen in the gas phase, kg/m^3^
*c_ig_*	mass concentration of *S_i_* species in the gas phase, kg/m^3^
*C_i_*	mass percentage of *S_i_* species in the liquid phase, wt%
*E_hi_*	activation energy for hydrogenation reaction of *S_i_* species, kJ/mol
*G_Vg_*	volumetric flow rate of gas phase, m^3^/h
*G_Vl_*	volumetric flow rate of liquid phase, m^3^/h
*G_Vrg_*	volumetric flow rate of recycled gas phase, m^3^/h
*k_dhi_*	dehydrogenation reaction rate constant of *S_i_* species, h^−1^
*k_hi_*	hydrogenation reaction rate constant of *S_i_* species, h^−1^
*k_hi_*_0_	pre-exponential factor of *S_i_* species in the Arrhenius Equation (13), h^−1^
*LHSV*	liquid hourly space velocity, h^−1^
*n_i_*	reaction order of *S_i_* species
*P*	pressure, Pa
*P_H_*_2_	partial pressure of hydrogen in the gas phase, Pa
*r_gl_*	gas/diesel volumetric ratio
*R*	universal gas constant, kJ/(molK)
*t*	temperature, °C
*t_ASTM_*	diesel ASTM 50% distillation temperature, °C
*T*	absolute temperature, K
*v_ri_*	reaction rate of *S_i_* species, kg/(m^3^h)
*V*	catalyst volume, m^3^
*Y_H_*_2__*g*_	molar percentage of hydrogen in the gas phase (H_2_ purity), mol%
*α*_1_	stoichiometric ratio between H_2_S and sulfur compounds
*α*_2_	stoichiometric ratio between NH_3_ and nitrogen compounds
*β*_1_	stoichiometric ratio between H_2_ and sulfur compounds
*β*_2_	stoichiometric ratio between H_2_ and nitrogen compounds
*β*_3_	stoichiometric ratio between H_2_ and aromatic compounds
*β*_4_	stoichiometric ratio between H_2_ and olefins
*η_i_*	hydrogenation degree of *S_i_* species
*ρ_l_*	density of liquid phase, kg/m^3^
*τ*	time, h
*τ_LHSV_*	residence time of the liquid phase in the catalyst bed (1/*LHSV*), h
Subscripts	
*e*	exit from the reactor
*g*	gas
*i*	compound species; *i* = 1 (or *S*) for sulfur compounds; *i* = 2 (or *N*) for nitrogen compounds; *i* = 3 (or *A*) for aromatic compounds; *i* = 4 (or *O*) for olefins
*l*	liquid
*m*	mean
*rg*	recycled gas
0	reactor inlet (*V* = 0)
