# Supplementary material for: Monitored and Predicted Data for a Diesel Fuel Hydrotreating Reactor
Source: Materials (Basel). 2025 May 25;18(11):2481. doi: 10.3390/ma18112481 (PMC12156934; doi:10.3390/ma18112481)
Supplement: Supplementary file 1 [file materials-18-02481-s001.zip › materials-3606433-supplementary.pdf]

materials

MDPI

Article

Monitored and Predicted Data for a Diesel Fuel Hydrotreating Reactor

Laura Elisabeta Petraş [1,2], Tănase Dobre [1,3,*], Nela Şerbănescu [2], Florian Daniel Pop [2] and Oana Cristina Pârvulescu [1,*]

1 Chemical and Biochemical Department, National University of Science and Technology POLITEHNICA Bucharest, 1-7 Gheorghe Polizu, 011061 Bucharest, Romania; laurapetras@yahoo.ro
2 Petromidia Refinery, 214 Năvodari Bld., 905700 Năvodari, Romania; nela.serbanescu@rompetrol.com (N.Ş.); floriandaniel.pop@rompetrol.com (F.D.P.)
3 Technical Sciences Academy of Romania, 26 Dacia Bld., 030167 Bucharest, Romania
* Correspondence: tghdobre@gmail.com (T.D.); oana.parvulescu@yahoo.com (O.C.P.)

Supplementary Materials

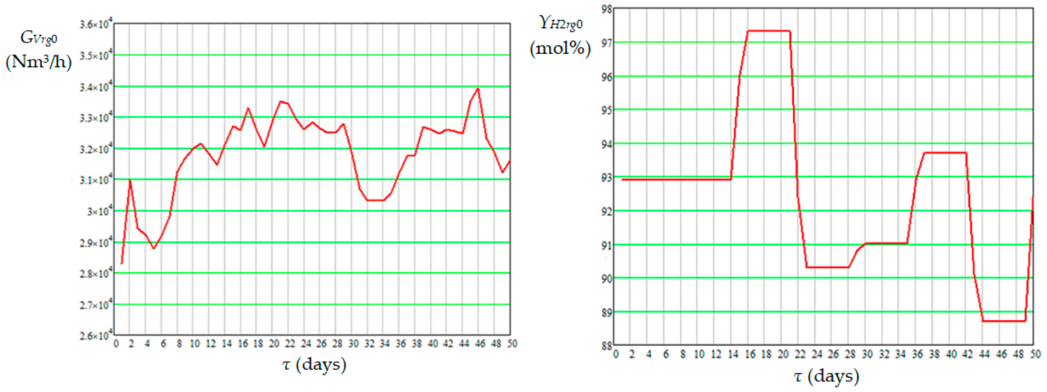

(**a**) (**b**)

Figure S1. Dynamics of volumetric flow rate of recirculated gas at the reactor inlet (a) and hydrogen purity of recirculated gas (b).

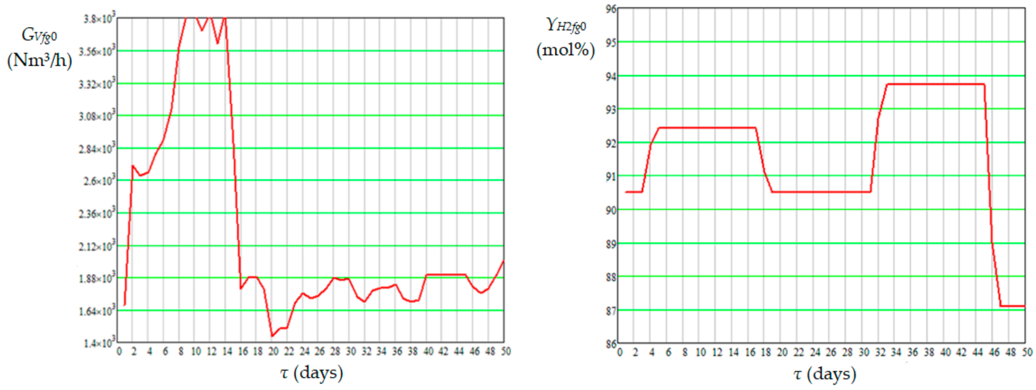

(**a**) (**b**)

Figure S2. Dynamics of volumetric flow rate of fresh gas at the reactor inlet (a) and hydrogen purity of fresh gas (b).

 https://doi.org/10.3390/xxxxx

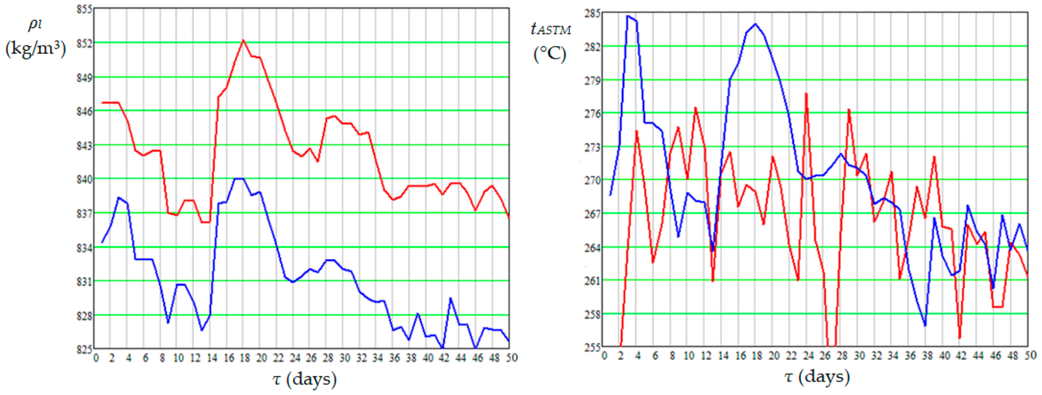

(**a**) (**b**)

Figure S3. Dynamics of diesel density (a) at the reactor inlet (in red) and outlet (in blue) and ASTM 50% distillation temperature (b) at the reactor inlet (in red) and outlet (in blue).
